# Regulation of ENaC-mediated alveolar fluid clearance by insulin via PI3K/Akt pathway in LPS-induced acute lung injury

**DOI:** 10.1186/1465-9921-13-29

**Published:** 2012-03-30

**Authors:** Wang Deng, Chang-Yi Li, Jin Tong, Wei Zhang, Dao-Xin Wang

**Affiliations:** 1 Department of Respiratory Medicine, Second Affiliated Hospital of Chongqing Medical University, 76 Linjiang Road, Yuzhong District, Chongqing 400010, China; 2 Department of Respiratory Medicine, First Affiliated Hospital of Chengdu Medical College, 278 Baoguang Road, Xindu District, Chengdu, Sichuan Province, China

**Keywords:** Alveolar fluid clearance, Akt, Epithelial sodium channel, Insulin, Phosphatidylinositol 3-kinase, Acute lung injury

## Abstract

**Background:**

Stimulation of epithelial sodium channel (ENaC) increases Na^+ ^transport, a driving force of alveolar fluid clearance (AFC) to keep alveolar spaces free of edema fluid that is beneficial for acute lung injury (ALI). It is well recognized that regulation of ENaC by insulin via PI3K pathway, but the mechanism of this signaling pathway to regulate AFC and ENaC in ALI remains unclear. The aim of this study was to investigate the effect of insulin on AFC in ALI and clarify the pathway in which insulin regulates the expression of ENaC in vitro and in vivo.

**Methods:**

A model of ALI (LPS at a dose of 5.0 mg/kg) with non-hyperglycemia was established in Sprague-Dawley rats receiving continuous exogenous insulin by micro-osmotic pumps and wortmannin. The lungs were isolated for measurement of bronchoalveolar lavage fluid(BALF), total lung water content(TLW), and AFC after ALI for 8 hours. Alveolar epithelial type II cells were pre-incubated with LY294002, Akt inhibitor and SGK1 inhibitor 30 minutes before insulin treatment for 2 hours. The expressions of α-,β-, and γ-ENaC were detected by immunocytochemistry, reverse transcriptase polymerase chain reaction (RT-PCR) and western blotting.

**Results:**

In vivo, insulin decreased TLW, enchanced AFC, increased the expressions of α-,β-, and γ-ENaC and the level of phosphorylated Akt, attenuated lung injury and improved the survival rate in LPS-induced ALI, the effects of which were blocked by wortmannin. Amiloride, a sodium channel inhibitor, significantly reduced insulin-induced increase in AFC. In vitro, insulin increased the expressions of α-,β-, and γ-ENaC as well as the level of phosphorylated Akt but LY294002 and Akt inhibitor significantly prevented insulin-induced increase in the expression of ENaC and the level of phosphorylated Akt respectively. Immunoprecipitation studies showed that levels of Nedd4-2 binding to ENaC were decreased by insulin via PI3K/Akt pathway.

**Conclusions:**

Our study demonstrated that insulin alleviated pulmonary edema and enhanced AFC by increasing the expression of ENaC that dependent upon PI3K/Akt pathway by inhibition of Nedd4-2.

## Introduction

Actue lung injury(ALI), the early stage of acute respiratory distress syndrome (ARDS), is a devastating clinical syndrome characterized by alveolar epithelial injury leading to non-cardiogenic pulmonary edema of flooding protein-rich fluid in the alveolar spaces with a mortality of approach 40%[1,2]. In vivo, alveolar fluid volume is determined by alveolar fluid clearance (AFC), the balance of transepithelial Na^+ ^transport [3]. AFC was impaired in ALI and removal of excessive alveolar edema fluid is an important way for effective treatment and better outcome[4,5].

It has been generally believed that epithelial sodium channel (ENaC) is the primary determinant of AFC, a driving force to remove edema fluid from alveolar spaces on the ion transport-dependent mechanism[6-8]. ENaC is composed of three homologous subunits, α, β and γ, which is expressed in a number of epithelial tissues including alveolar epithelial cells [9,10]. Unable to clear alveolar edema fluid, α-ENaC gene knock-out mice died within 40 hours after birth [11].β-ENaC gene in alveolar epithelium was proved to be required for AFC in mice [12]. The mice lacking γ-ENaC gene influenced the alveolar edema fluid absorption that was essential for AFC [13]. Thus, the three subunits of ENaC play a key role in AFC.

The phosphatidylinositol 3-kinase (PI3K) family, divided into IA, IB, II, and III classes, consists of a catalytic domain and a regulatory domain and participates cell responses including cell survival, metabolism,gene expression,vesicular trafficking, cytoskeletal rearrangement and migration [14,15]. Insulin increases Na^+ ^transport by trafficking ENaC subunits to the apical membrane in kidney cells via PI3K-dependent mechanism [16,17]. PI3K has been identified as integral for regulation of ENaC by insulin [18]. It is well established that insulin activates PI3K by linking to the insulin receptor and generating phosphatidylinositol-3,4,5-triphosphate to promote the activation of protein kinase B(Akt), an important downstream kinase that regulates glycogen and protein synthesis [19,20]. Upon insulin stimulation, the pleckstrin homology domain of Akt binds to lipid messengers and is phosphorylated at Thr308 and Ser473 by recruition to the plasma membrane [21]. However, how this signaling pathway transduction converge to regulate AFC and three subunits of ENaC in ALI has not yet been elucidated.

In this study, we aimed to investigate the effect of insulin on AFC and the expression of ENaC via PI3K/Akt pathway in vitro and in vivo. We found that insulin attenuated lung injury in LPS-induced ALI, alleviated pulmonary edema and enhanced AFC by increasing the expression of ENaC that dependent upon PI3K/Akt pathway by inhibition of Nedd4-2.

## Methods

### Materials

Male Sprague-Dawley rats weighing 200-250 g (Department of Laboratory Animal Center, Chongqing Medical University) were housed under specific pathogen-free conditions in a temperature- and humidity-controlled environment and given free access to food and water with the Guide for the Care and Use of Laboratory Animals. Reagents for cell culture were provided by the Institute of Life Science, Chongqing Medical University. Lipopolysaccharide (LPS, Escherichia coli serotype O111:B4), LY294002 (PI3K inhibitor [22]), wortmannin (PI3K inhibitor [22]), amiloride (sodium channel inhibitor), sodium pentobarbital and Evans blue were purchased from Sigma (St Louis, MO, USA).

Akt inhibitor (1 L-6-hydroxymethyl-chiroinositol2 [(R)-2-Omethyl-3-O-octadecylcarbonate]) was purchased from Enzo Life Sciences (Farmingdale, NY, USA).

Serum- and glucocorticoid-regulated protein kinase1 (SGK1) inhibitor (2-Cyclopentyl-4-(5-phenyl-1H-pyrrolo [2,3-b]pyridin-3-yl-benzoic acid) was purchased from Tocris bioscience(Bristol, UK). Rabbit anti- α-ENaC,β-ENaC and γ-ENaC antibodies were purchased from Santa Cruz Biotechnology(Santa Cruz, CA, USA) Rabbit anti- Phospho-Akt (Ser473) and total Akt monoclonal antibodies were obtained from Cell Signaling Technology (Beverly, MA, USA). Rabbit anti-Nedd4-2 polyclonal antibody was purchased from ABcam (Cambridge, MA, USA).

The study was approved by the Ethics Committee of the Second Affiliated Hospital of Chongqing Medical University.

### Animal model and intervention

Rats were anesthetized by intraperitoneal administration of sodium pentobarbital (50 mg/kg). ALI model was established by LPS (5.0 mg/kg) with intraperitoneal injection followed by insertion of an internal jugular vein catheter for drug administration. Human Insulin (Humulin 70/30; Eli Lilly, Indianapolis, IN, USA) was administered at a dose of 0.1 U/kg/h and at a rate of 2.5 mU/h/rat via micro-osmotic pumps(Zhejiang University Medical Instrument Co. Ltd., Hangzhou, China) 16 hours before LPS exposure. Wortmannin (0.06 mg/kg) were injected retro-orbitally three times at -90, +90, and +360 minutes relative to the LPS injection. Rats in control group were received an equivalent volume of saline. Rats were killed 8 hours after LPS or saline treatment. Blood samples, bronchoalveolar lavage fluid (BALF) and lung tissue were obtained for analysis.

### Cell isolation, culture and treatment

Alveolar epithelial type II (ATII) cells were isolated from male Sprague-Dawley rats by elastase digestion of lung tissue and then differentially adhered on IgG-coated plates as previously described [23]. Purity of the ATII cells were determined by microscopic analysis, indicative of epithelial cell lineage and by immunohistochemistry for surfactant protein-C, indicative of ATII cell. ATII cells were seeded onto plastic culture dishes and cultured in a 5% CO2, 95% air atmosphere in DMEM containing 10% fetal bovine serum, 100 U/ml penicillin and 0.1 mg/ml streptomycin after isolation. On day 3 after isolation, the cells were pre-incubated with LY294002 (10 μM), Akt inhibitor(100 nM) and SGK1 inhibitor(10 μM) for 30 minutes before insulin(200 mU/L) treatment for 2 hours and the experiments were performed.

### Measurement of glucose and insulin levels

Blood samples were withdrawn from the catheter by centrifuging at 3000 rpm at 4°C for 15 minutes. Glucose levels in the plasma were analyzed by Glucometer OneTouch (Johnson& Johnson Medical Ltd., Shanghai, China). Human insulin levels in the plasma were analyzed by a ELISA kit for only human insulin (10-1132-01; Mercodia, Uppsala, Sweden). Total insulin levels in the plasma were analyzed by a ELISA kit for rat plus human insulin (10-1251-01; Mercodia).

### Measurement of TNF-α, IL-6, protein levels and myeloperoxidase assay in bronchoalveolar lavage fluid

BALF was performed on the right lung lavaged with 0.9% NaCl (5 ml) at room temperature and was collected after infusion with six times. More than 90% of BALF was collected from each animal and was centrifuged at 14,000 rpm for 30 minutes at 4°C to remove cell debris. The supernatant from the first two washes was pooled and analyzed for total protein. The rest of BALF was stored at -80°C for tumor necrosis factor-α(TNF-α), interleukin-6(IL-6), and myeloperoxidase (MPO) analysis. The total cell counts were determined by a hemocytometer and differential cell counts were assessed on cytocentrifuge preparations stained with Diff-Quik (Sigma, St. Louis, MO, USA). The measurement of TNF-a, IL-6 were analyzed by Enzyme-linked immunosorbent assay (ELISA) kits (R&D Systems, Minneapolis, MN, USA). Total protein levels were determined by a protein assay kit (KeyGEN, KeyGEN Bio TECH Co., Nanjing, China). MPO activity, an indicator of neutrophil activation [24], was determined by a MPO assay kit (Nanjing Jiancheng Bioengineering Institute, Nanjing, China). All assays were done according to the manufacturer's instructions.

### Lung histology evaluation

The left lower lung lobes were harvested and fixed in 10% neutral buffered formalin for 24 hours. Then they were embedded in paraffin and stained with hematoxylin and eosin (H&E) for microscope observation. A semi-quantitative scoring system was adopted to evaluate the lung injury including intraalveolar exudate, interstitial edema, alveolar hemorrhage, and inflammatory cell infiltration [25]. The grading scale of pathologic findings was used in a light microscope:0 = no injury;1 = slight injury(25%);2 = moderate injury(50%); 3 = severe injury(75%); and4 = very severe injury (almost 100%).

### Immunocytochemistry

The paraffin was dewaxinged with Xylene and hydrated with ethanol, and then it was treated with 3%H_2_O_2 _to inhibit endogenous peroxidase activity for 10 minutes and rinsed with phosphate buffer solution (pH 7.6). It was blocked with bovine serum albumin for 30 minutes and incubated with primary antibodies at 4°C for 24 hours. Then, biotinylated anti-rabbit IgG (Santa Cruz Biotechnology) was reacted for 30 minutes in an incubator at 37°C. After washing with phosphate buffer solution for three times, it was reacted with avidin-biotin-peroxidase complex (Sigma) for 30 minutes and then stained with DAB (Sigma), a colouring agent, for 5 minutes. For control staining, it was also reacted with hematoxylin for 30 seconds. Normal rabbit isotype IgG (Santa Cruz Biotechnology) was a substitute for the primary antibodies in the above process as a negative control. The number of positive cells was counted in randomly 5 high-power fields (magnification 400 ×) of each section and averaged with a light microscopy.

### Measurement of total lung water content and alveolar fluid clearance

Total lung water content (TLW), a quantification of pulmonary edema, was measured as previously described[26]. The left lung was isolated for determination of TLW. The lung was weighed in an automatic electric balance (Sartorius, Goettingen, Germany), then placed in an oven at 80°C for 48 hours and weighed again to obtain its dry weight. TLW was calculated as follows: TLW = (wet lung weigh-dry lung weight)/(dry lung weight).

AFC was measured according to the established procedure [27]. Briefly, the isolated right lung was placed in a humidified incubator at 37°C and ventilated with 100% nitrogen to remove oxygen from the alveolar spaces. Physiological saline solution (5 ml/kg) containing 5% albumin and Evans blue dye (0.15 mg/ml) was injected into the alveolar spaces at an airway pressure of 7 cm H_2_O. Alveolar fluid was aspirated 1 h after instillation. The concentrations of Evans blue-labeled albumin in the injected and aspirated solutions were measured by a spectrophotometer (Beckman Coulter, Los Angeles, CA, USA). AFC was calculated as follows:

$$\text{AFC = [(}{\text{V}}_{\text{i}}\text{ - }{\text{V}}_{\text{f}}\text{)/}{\text{V}}_{\text{i}}\text{]}\times \text{100\% }\;{\text{V}}_{\text{f}}=\left({\text{V}}_{\text{i}}-{\text{P}}_{\text{i}}\right)/{\text{P}}_{\text{f}}$$

V represents the injected volume (i) and final volume (f) of alveolar fluid. P represents the injected (i) and final (f) concentration of Evans blue-labeled 5% albumin solution.

### RNA extraction and Reverse Transcription Polymerase Chain Reaction (RT-PCR) analysis

Total RNA was extracted from the lung tissue and cells with a RNA extraction kit (TaKaRa, Japan), according to the manufacture's instructions. The concentration and purity of RNA were estimated on a spectrophotometer. Primer sequences for α-,β-, and γ-ENaC were used for PCR amplification: α-ENaC(509 bp), 5'- TACCCT TCCAAG TATACACAGC-3' (forward) and 5'- CAGAAGGAGACTCCGAATTAGT-3'(reverse);β-ENaC(406 bp), 5'-GCTAAAGAGCTAGCAG TAATGG-3'(forward)and5'-CTGGTGTTTGTTATGCCTAGAG-3'(reverse);γ-ENaC(363 bp), 5'- GGATCCTGAGAGAGAATCATGC-3'(forward)and5'-GTGTCCAGCTATGCCCTTTAAC-3' (reverse);β-actin(871 bp), 5'-GTACAACCTTCTTGCAGCTCCT-3'(forward)and5'-ACAGGATT CCATACCCAGGAAG -3' (reverse). Two-step RT-PCR Kit (TaKaRa, Japan) was used for reverse transcription with PCR amplification analyzer(Eppendorf, Hamburg, Germany). Reverse transcription reaction conditions were 65°C for 5 minutes, 42°C for 30 minutes, 95°C for 5 minutes and4°C for 5 minutes. Polymerase chain reactions comprised pre-denaturation at 94°C for 60 seconds, 30 cycles of denaturation at 94°C for 30 seconds, annealing at 53°C (α-ENaC), 53°C (β-ENaC), 55°C (γ-ENaC) and 55°C (β-actin) for 30 seconds and polymerization at 72°C for 60 seconds. Each PCR product was run on a 1.0% agarose gel containing ethidium bromide and was visualized with Gel Imaging System (Bio-Rad, Hercules, CA, USA).

### Western blotting analysis and immunoprecipitation

Proteins were obtained with 1 ml of lysis buffer and 1 ml of extraction buffer by using a protein extraction kit(KeyGEN) according to the manufacture's instructions and stored at -80°C for analysis. Proteins were separated by 10% SDS-PAGE and transferred to polyvinylidene fluoride menbranes. After blocking with 5% nonfat dried milk in Tris-buffered saline containing 0.05% Tween 20, the membranes were incubated with primary antibodies α-, β-, γ-ENaC(1:300), p-AK(1:1000), Akt (1:1000), β-actin(1:500) and Nedd4-2(1:1000) overnight at 4°C, and then reacted with horseradish peroxidase-conjugated secondary antibody (1:5000)(Santa Cruz Biotechnology) at room temperature for 1.5 hours. Using a Western Blot Enhanced Chemiluminescence (ECL) method, the protein bands were visualized by UVP Gel imaging system(Upland, CA, USA) and analyzed by Labworks software(version 4.6). 500 μg of total proteins were immunoprecipitated from cell lysates with the indicated antibodies at 4°C overnight with rotation and then incubated with 40 μl of protein A/G-agarose (Santa Cruz Biotechnology) beads for 4 hours at 4°C with rotation. Beads were washed four times with lysis buffer and resuspended in sample buffer. Samples were subjected to SDS-PAGE and transferred to polyvinylidene fluoride membranes followed by western blot analysis for Nedd4-2.

### Statistical analysis

All data were described as mean ± S.E.M.. Statistical analysis was performed by Student's *t*-test and one-way analysis of variance (ANOVA) using SPSS 12.0 software (SPSS Inc, Chicago, USA).*P *value < 0.05 was considered statistically significant.

## Results

### Effect of exogenous insulin on plasma insulin and glucose levels

**Figure 1:**
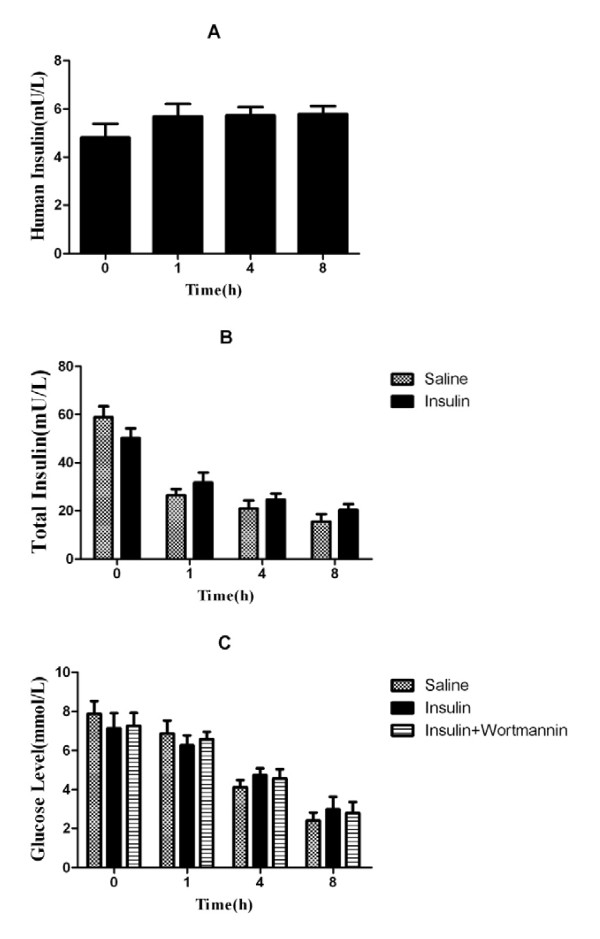
**Human insulin (A), total insulin (rat + human) (B), and plasma glucose level (C) were measured by ELISA (insulin) or glucometer (glucose) at 0, 1, 4, 8 hours in normal and LPS-induced actue lung injury rats (n = 10 per group)**. Data are presented as mean ± S.E.M.

Insulin at a dose of 0.1 U/kg had no effect on plasma glucose levels in rats (Table 1). Micro-osmotic pumps were continuously infused throughout the experimental period at a rate of 2.5 mU/h/rat. Human insulin levels were maintained at a constant level in insulin-treated rats during LPS-induced ALI (Figure 1.A). There was no significant difference in total insulin levels between insulin-treated and saline-treated rats during LPS-induced ALI (Figure 1.B). Plasma glucose levels showed no significant difference at 0, 1, 4, 8 hours after LPS-induced ALI between insulin-treated and saline-treated rats (Figure 1.C). Also, wortmannin at a dose of 0.06 mg/kg had no effect on plasma glucose levels in our study, which indicated that insulin treatment did not exacerbate LPS-induced hypoglycemia (Figure 1.C).

**Table 1:** Effect of exogenous insulin on plasma glucose levels in rats

Insulin dose(0.1 U/kg)					
Time after insulin(min)	0	30	60	120	240

Plasma glucose(mmol/L)	4.7 ± 0.5	5.0 ± 0.4	4.8 ± 0.7	5.1 + 0.3	5.2 + 0.3

Data are presented as mean ± S.E.M (n = 5 per group).

### Effect of exogenous insulin on TNF-α, IL-6, BALF protein, and neutrophil infiltration in LPS-induced actue lung injury

**Figure 2:**
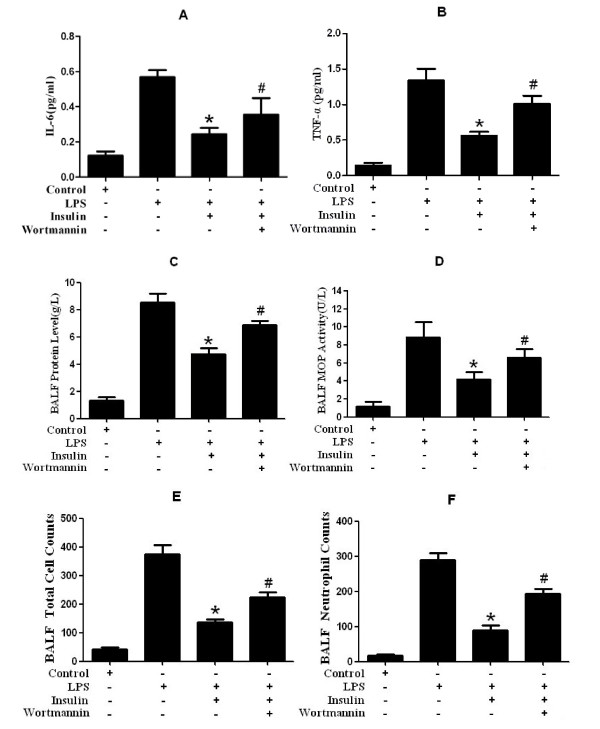
**Measurement of TNF-α(A), IL-6(B), protein(C), MOP activity (D), total cell counts(E), and neutrophil counts(F) in Bronchoalveolar lavage fluid (BALF) 8 hours after LPS-induced actue lung injury or saline treatment (n = 5 per group)**. Data are presented as mean ± S.E.M.*P < 0.05 vs LPS group;# *P *< 0.05 vs LPS + Insulin group.

Insulin significantly reduced LPS-induced increase in TNF-α, IL-6, protein level, MPO activity, total cell counts, and neutrophil counts in BALF. (*p *< 0.05, Figure 2. A-F). However, the effects of insulin were significantly blocked by wortmannin (*p *< 0.05, Figure 2. A-F).

### Exogenous insulin attenuated lung injury in LPS-induced actue lung injury

**Figure 3:**
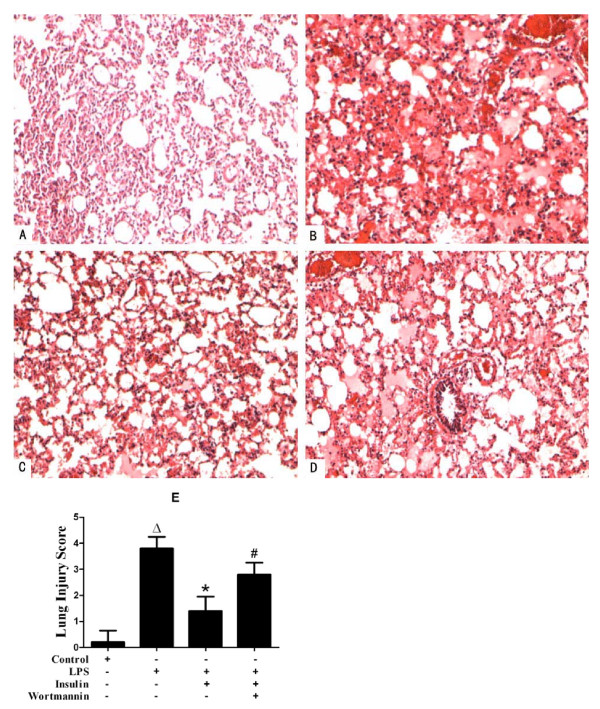
**Effect of exogenous insulin on the morphology of lung 8 hours after LPS-induced actue lung injury or saline treatment**. (A) Control group: No histologic changes were observed. (B) LPS group: Marked intraalveolar exudate, edema, and inflammatory cell infiltration in the interstitial and alveolar spaces. (C) LPS + insulin group: Insulin significantly attenuated lung injury. (D) LPS + insulin + wortmannin group: Wortmannin significantly blocked the effect of insulin in the lung pathology. (E) Lung injury score: Insulin significantly reduced ALI-induced increase in lung injury score. Wortmannin significantly blocked the effect of insulin(n = 5 per group). Original magnification: 100 ×. Data are presented as mean ± S.E.M.Δ*P *< 0.05 vs Control group;*P < 0.05 vs LPS group;# *P *< 0.05 vs LPS + Insulin group.

The lung tissue was significantly injured with the presence of intraalveolar exudate, edema, and inflammatory cell infiltrationin LPS group compared with that in control group, as an evidence by an increase in lung injury score(*p *< 0.05, Figure 3A, B, E). Insulin significantly attenuated LPS-induced pathologic changes by the evidence of a decrease in lung injury score (*p *< 0.05, Figure 3C, E). Coadministration of wortmannin significantly blocked the effect of insulin (*p *< 0.05, Figure 3D, E).

### Effect of exogenous insulin on pulmonary edema and alveolar fluid clearance in LPS - induced actue lung injury

**Figure 4:**
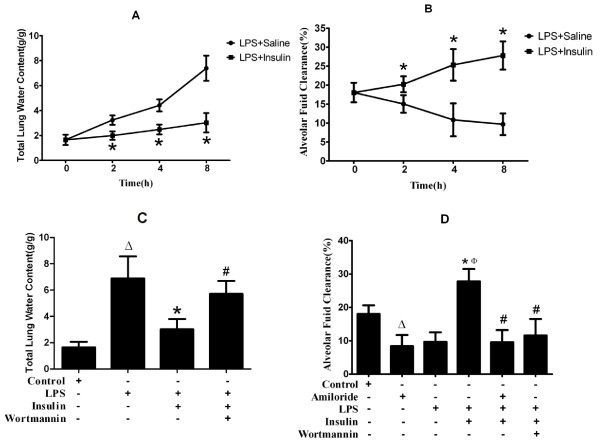
**Effect of exogenous insulin on pulmonary edema and alveolar fluid clearance in LPS-induced actue lung injury (n = 6 per group)**. (A) Total lung water content at 0, 2, 4, 8 hours after LPS or insulin treatment. (B) Alveolar fluid clearance at 0, 2, 4, 8 hours after LPS or insulin treatment. (C) Total lung water content at 8 hours after LPS-induced actue lung injury or saline treatment (D) Alveolar fluid clearance at 8 hours after LPS-induced actue lung injury or saline treatment. Albumin solution containing amiloride (5 × 10^-4 ^M) were injected into the alveolar spaces. Data are presented as mean ± S.E.M.Δ*P *< 0.05 vs Control group;Φ*P *< 0.05 vs Amiloride group;*P < 0.05 vs LPS group;# *P *< 0.05 vs LPS + Insulin group.

TLW was significantly decreased and AFC was significantly increased by insulin treatment after LPS-induced ALI at 2, 4, 8 hours(*p *< 0.05, Figure 4 A, B). Insulin-induced decrease in TLW was significantly blocked by wortmannin 8 hours after LPS-induced ALI (*p *< 0.05, Figure 4 C). AFC was significantly increased by 40% with insulin treatment, but was significantly decreased by 35% with wortmannin in LPS - induced ALI (*p *< 0.05, Figure 4 D). Also, amiloride, a sodium channel inhibitor, significantly decreased insulin-induced increase in AFC by 47% (*p *< 0.05, Figure 4 D).

### Effect of exogenous insulin on lung localization of ENaC in LPS-induced actue lung injury

**Figure 5:**
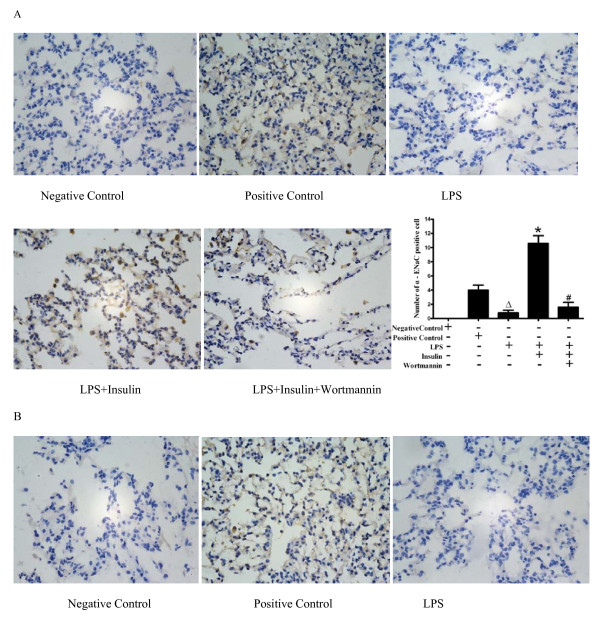
**Effect of exogenous insulin on the protein expression of α- ENaC(A), β- ENaC(B), and γ-ENaC(C) in rat lung 8 hours after LPS-induced actue lung injury or saline treatment**. The number of positive cells was counted in randomly 5 high-power fields of each section and averaged (five sections from 5 rats in each group). The number of cells expressing α-, β-, and γ-ENaC were significantly decreased in LPS-induced actue lung injury, compared with those in positive control group. However, the number of cells expressing α-, β-, and γ-ENaC were strongly increased by insulin treatment, but were decreased by wortmannin. Brown immunostained cell was positive (original magnification 400 ×).Δ*P *< 0.05 vs Positive control group;*P < 0.05 vs LPS group;# *P *< 0.05 vs LPS + Insulin group.

Immunohistochemical analysis was used to determined the lung distribution of α-, β-, and γ-ENaC in rat lung 8 hours after LPS or saline treatment. Positively immunostained cells appeared brown. The expressions of α-, β-, and γ-ENaC were specifically localized to the alveolar epithelium. The number of cells expressing α-, β-, and γ-ENaC were significantly decreased in LPS-induced actue lung injury, and were strongly increased by insulin treatment, but were decreased by wortmannin (Figure 5).

### Exogenous insulin increased the expression of alveolar epithelial sodium channel in vivo and in vitro

**Figure 6:**
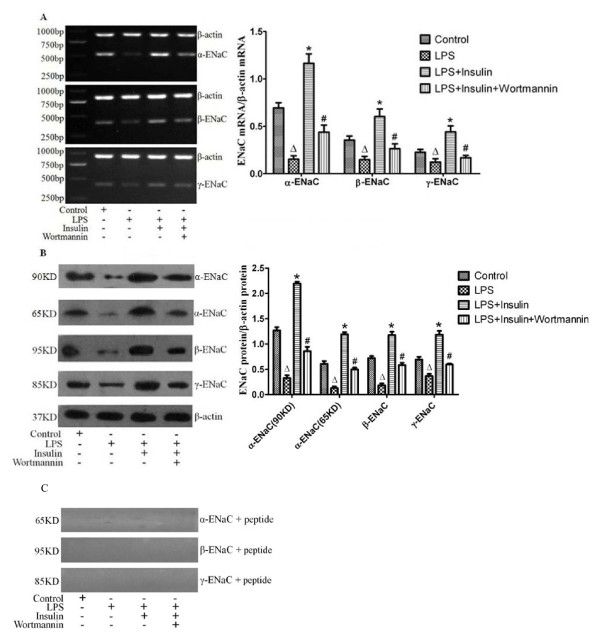
**Expressions of alveolar epithelial sodium channel(α-ENaC, β-ENaC and γ-ENaC) in rat lung were measured by RT-PCR(A) and western blotting(B) 8 hours after LPS-induced actue lung injury or saline treatment (n = 5 per group)**. Proteins using the same antibodies against α-ENaC, β-ENaC and γ-ENaC plus blocking peptides specific for these antibodies were re-blotted(C). Data are presented as mean ± S.E.M.Δ*P *< 0.05 vs Control group;*P < 0.05 vs LPS group;# *P *< 0.05 vs LPS + Insulin group.

**Figure 7:**
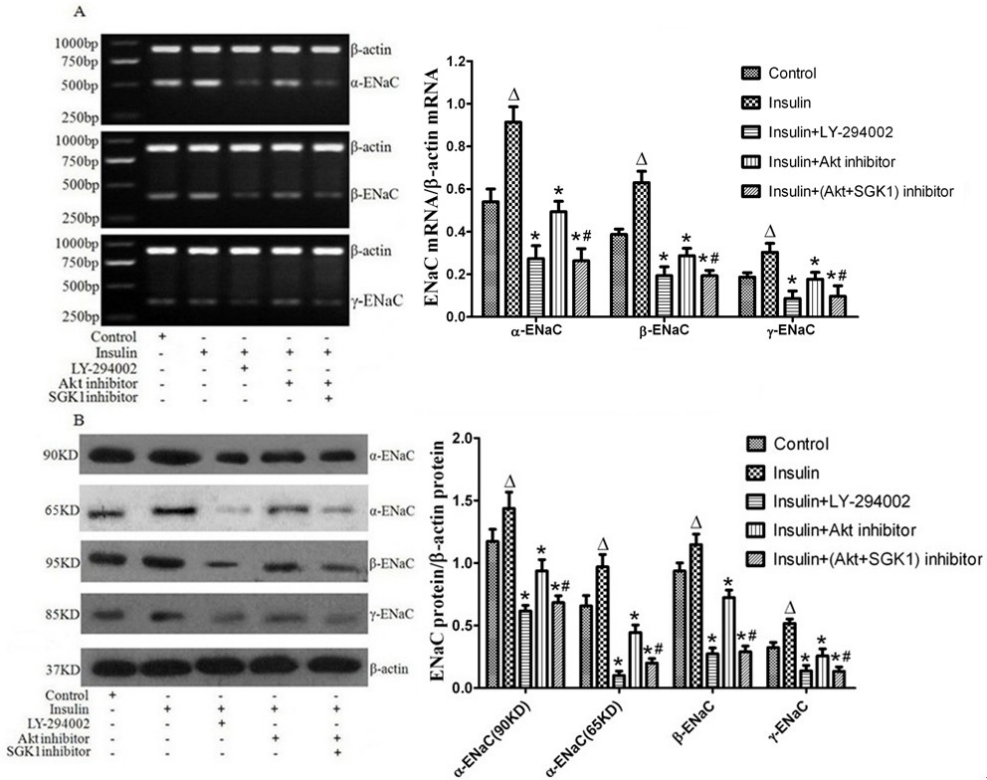
**Expressions of alveolar epithelial sodium channel(α-ENaC, β-ENaC and γ-ENaC) in alveolar epithelial type II cells pretreated with LY294002(10 μM), Akt inhibitor(100 nM) and SGK1 inhibitor(10 μM) for 30 minutes before insulin treatment for 2 hours were measured by RT-PCR(A) and western blotting(B)**. Proteins using the same antibodies against α-ENaC, β-ENaC and γ-ENaC plus blocking peptides specific for these antibodies were re-blotted(C). Data are presented as mean ± S.E.M.Δ*P *< 0.05 vs Control group;*P <# 0.05 vs Insulin group; *P *< 0.05 vs Insulin + Akt inhibitor group.

To clarify the effect of insulin on AFC mediated by ENaC, the expressions of α-, β- and γ-ENaC were measured by RT-PCR and western blotting respectively. Two forms (90 kDa and 65 kDa) of α-ENaC were detected by western blotting (Figure 6 B; Figure 7 B). In vivo, the mRNA and protein expression levels of α-, β- and γ-ENaC in rat lung showed significant increases by insulin treatment 8 hours after LPS-induced ALI (*P *< 0.05, Figure 6 A, B), but the mRNA and protein expression levels of three ENaC subunits were significantly decreased with the administration of wortmannin compared with those by insulin treatment (*P *< 0.05, Figure 6 A, B). In vitro, the mRNA and protein expression levels of α-, β- and γ-ENaC were significantly increased by insulin treatment for 2 hours in ATII cells (*P *< 0.05, Figure 7A, B), but pretreatment with LY294002 and Akt inhibitor prevented the insulin-induced increase in the mRNA and protein expression levels of α-, β- and γ-ENaC in ATII cells respectively (*P *< 0.05, Figure 7A, B). In addtion, the mRNA and protein expression levels of α-, β- and γ-ENaC in ATII cells with co- administration of Akt inhibitor and SGK1inhibitor showed the similar changes compared with those by LY294002 treatment(*P *> 0.05, Figure 7A, B), and were significantly decreased compared with those by Akt inhibitor treatment (*P *< 0.05, Figure 7A, B). The bands were absent when proteins were blotted with the α-ENaC, β-ENaC and γ-ENaC antibodies in the presence of the blocking peptide both in vivo(Figure 6C) and in vitro(Figure 7 C). These results indicated that insulin-induced expression of ENaC by Akt phosphorylation via activating PI3K pathway.

### Exogenous insulin activated the P13K/Akt pathway and inhibited Nedd4-2 in vivo and in vitro

**Figure 8:**
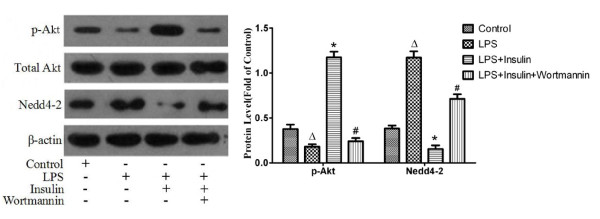
**Expressions of phosphorylated Akt and Nedd4-2 in rat lung 8 hours after LPS-induced actue lung injury or saline treatment were measured by western blotting**. The band intensity of phosphorylated Akt and Nedd4-2 were quantitated by normalized for total Akt and β-actin respectively and expressed as fold of the control (n = 3 per group). Data are presented as mean ± S.E.M.Δ*P *< 0.05 vs Control group;*P < 0.05 vs LPS group;# *P *< 0.05 vs LPS + Insulin group.

**Figure 9:**
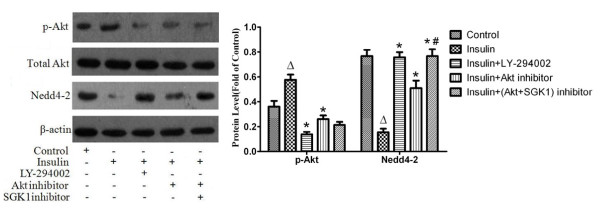
**Expressions of phosphorylated Akt and Nedd4-2 in alveolar epithelial type II cells pretreated with LY294002(10 μM), Akt inhibitor(100 nM) and SGK1 inhibitor(10 μM) for 30 minutes before insulin treatment for 2 hours were measured by western blotting**. The band intensity of phosphorylated Akt and Nedd4-2 were quantitated by normalized for total Akt and β-actin respectively and expressed as fold of the control. Data are presented as mean ± S.E.M.Δ*P *< 0.05 vs Control group;*P < 0.05 vs Insulin group; # *P *< 0.05 vs Insulin + Akt inhibitor group.

**Figure 10:**
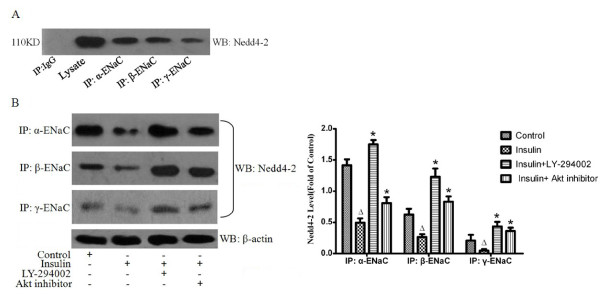
**Association of Nedd4-2 with α-ENaC, β- ENaC and γ-ENaC**. (A) Protein from alveolar epithelial type II cells lysate was immunoprecipitated with rabbit anti- α-ENaC, β-ENaC and γ-ENaC antibodies or with goat anti-rabbit IgG. Levels of coimmunoprecipitated Nedd4-2 were detected by Western blotting (WB) with an anti-Nedd4-2 antibody. The IgG did not immunoprecipitate Nedd4-2, thus serving as a negative control.(B) Effects of insulin, LY294002 and Akt inhibitor on Nedd4-2 in coimmunoprecipitated α-ENaC, β- ENaC and γ-ENaC. Levels of coimmunoprecipitated Nedd4-2 were significantly decreased by insulin treatment. LY294002 and Akt inhibitor significantly blocked the decrease in coimmunoprecipitated Nedd4-2 induced by insulin. Data are normalized for the amount of Nedd4-2 immunoprecipitated and expressed as fold of the control. Data are presented as mean ± S.E.M.Δ*P *< 0.05 vs Control group;*P < 0.05 vs Insulin group.

To further investigate whether regulation of ENaC by insulin via PI3K/Akt pathway, the level of Ser^473^-phosphorylated Akt, a reliable residue to read out of PI3K activity[28], and Nedd4-2, a binding site for regulation of ENaC function[29], were measured by western blotting and immunoprecipitation. The protein level of phosphorylated Akt was markedly increased in rat lung by insulin treatment 8 hours after LPS-induced ALI(*P *< 0.05, Figure 8). Wortmannin abolished the insulin-induced increase in the protein level of phosphorylated Akt (*P *< 0.05, Figure 8). However, the protein level of Nedd4-2 was significantly decreased by insulin treatment and was significantly increased by co-administration of wortmannin and insulin (*P *< 0.05, Figure 8). In ATII cells pretreated with LY-294002 and Akt inhibitor respectively, insulin-induced increase in the protein levels of phosphorylated Akt were markedly decreased (*P *< 0.05, Figure 9). The level of phosphorylated Akt in ATII cells was also significantly blocked by co- administration of Akt inhibitor and SGK1inhibitor compared that in cells treated with insulin (*P *< 0.05, Figure 9). In a contrast, the protein levels of Nedd4-2 were markedly higher in cells pretreated with LY-294002, Akt inhibitor and Akt inhibitor plus SGK1inhibitor compared with those in cells treated with insulin respectively (*P *< 0.05, Figure 9). Western blot analysis of α-, β- and γ-ENaC immunocomplexes with anti-Nedd4-2 antibody identified a band that was the same size as the one observed with ATII cells lysate and no such band was observed with control IgG, which showed Nedd4-2 interacted with α-, β- and γ-ENaC in cells under basal conditions(Figure 10A). The inhibitory effect of insulin on the levels of Nedd4-2 immunoprecipitated in α-, β- and γ-ENaC were significantly abolished by LY-294002 and Akt inhibitor repectively(Figure 10B). These findings strongly indicated that the down-regulation of Nedd4-2 that interacted with ENaC by insulin via P13K/Akt pathway.

### Exogenous insulin decreased mortality of rats in LPS-induced actue lung injury

**Figure 11:**
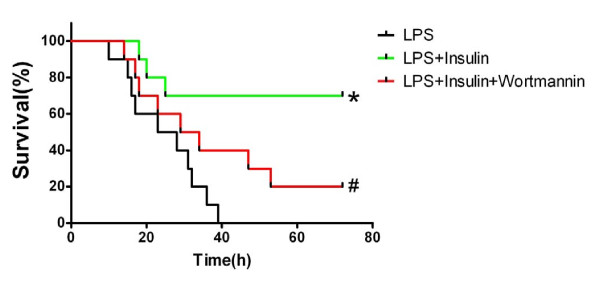
**Effect of exogenous insulin on survival of LPS - induced actue lung injury in rats**. Insulin or saline (LPS group) was administered continuously by a micro-osmotic pump. Survival was evaluated for 72 hours. Results are presented as a Kaplan-Meier plot (n = 10 per group).*P < 0.01 vs LPS group;# *P *< 0.05 vs LPS + Insulin group.

Insulin treatment significantly improved the survival of rats with ALI (*P *< 0.01, Figure 11), but wortmannin significantly inhibited the survival of rats treated with insulin in LPS-induced ALI (*P *< 0.05, Figure 11).

## Discussion

In the present study, our results demonstrated insulin played an therapic role in LPS-induced ALI and the mechanism of ENaC-mediated AFC by which insulin activated PI3K/Akt signaling pathway in vitro and in vivo. Results from animal and human studies indicated the hyperglycemia was associated with exacerbation of inflammation and promotion of injury in ALI and insulin treatment while maintaining euglycemia was found to attenuate the inflammatory response, reduce lung injury, and decrease the morbidity [30-34]. LPS models the effects of Gram-negative bacteria to induced ALI in animals and humans as a common methodology [35,36]. Therefore, a model of ALI with non-hyperglycemia and continuously infused human insulin by micro-osmotic pumps at a dose and a rate that maintained the glucose levels within normal range and did not worsen LPS-induced hypoglycemia were used in this study. Also, the dose of human insulin infused in the study would just only have an effect of anti-inflammatory mechanism rather than modulation of glucose metabolism that previously reported [37]. Insulin-induced phosphorylation of Akt in the liver was not observed by the low dose in our pre-experiment, which may also explain the tissue-specific difference in the activation of PI3K/Akt pathway by insulin to have an effect on inflammatory response without affecting glucose levels. The effect of wortmannin did not completely block the effect of insulin according to our results, which may be due to the possibility that additional mechanisms also contribute to the effects of insulin [38].

LPS stimulates macrophages, neutrophils, and other immune cells to produce different mediators including cytokines such as TNF-α, IL-6 that recruits polymorphonuclear neutrophils into the injured site and contribute to the pathogenesis of ALI and ARDS [39,40]. Activated neutrophils release various kinds of mediators, and secrete MPO enzyme, an indicator of neutrophil accumulation in tissues by its activity [24], are recognized to be a primary mechanism in the development of ALI [41]. In the current study, insulin inhibited LPS-induced increase in TNF-α, IL-6, neutrophil counts and MPO activity in BALF. Wortmannin, a PI3K inhibitor, abolished the insulin-induced reduction in TNF-α, IL-6, neutrophil counts and MPO activity produced by LPS and insulin-induced phosphorylation of Akt, which indicated the inhibition of PI3K/Akt pathway. The results were consistent with previous studies that illustrated PI3K/Akt signaling pathway played an important role as a negative regulation of LPS-induced acute inflammatory responses in vitro and in vivo[42-44]. In addition, activated neutrophils transmigrated across the endothelial surface into lung by release of reactive oxygen species, resulting in alveolar capillary barrier leakage, interstitial and alveolar edema after adhering to lung endothelium [45]. In this study, insulin attenuated LPS-induced ALI by evaluation of pulmonary edema and protein leakage in the alveolar spaces, histologic lung injury score, and survival rate. Wortmannin blocked the effects of insulin on LPS-induced ALI, which showed the involvement of PI3K/Akt pathway. These data indicated that activation of PI3K/Akt pathway by insulin contributed to attenuation of lung injury in ALI.

Pulmonary edema accumulates as a consequence of changes in hydrostatic pressure gradients or increased alveolar capillary permeability. It is well accepted that AFC, a process to remove edema fluid from the alveolar spaces, is of particular importance by Na^+ ^reabsorption from the alveolar spaces via ENaC in ALI/ARDS [8]. In the present study, insulin enhanced AFC that resulted in the decrease of pulmonary edema in LPS-induced ALI, which was consistent with the finding that increase in AFC could decrease the lung water volume [27]. Also, as demonstrated by the present experiment, amiloride, a sodium channel inhibitor, ininhibited AFC stimulated by insulin, supporting the stimulatory effect of insulin on AFC via ENaC in ALI, which was in agreement with previous study reporting that a lower AFC in a mouse model of type 2 diabetes was mainly due to decreased active Na^+ ^transport by ENaC [46]. Meawhile, AFC stimulated by insulin was significantly decreased by wortmannin indicated that PI3K was essential for the maintenance of Na^+ ^absorption previously reported[47]. Therefore, the link between ENaC and PI3K signaling pathway was further investigated in our study. In vivo, the expressions of α-, β- and γ-ENaC and the level of phosphorylated Akt were increased by insulin but were decreased by wortmannin in LPS-induced ALI. LY294002, a PI3K inhibitor, markedly prevented insulin-induced expressions of α-, β- and γ-ENaC and the level of phosphorylated Akt, which were consistent with the results in vivo. Also, Akt inhibitor, reported to inhibit Akt [48], blocked the expressions of α-, β- and γ-ENaC and the level of phosphorylated Akt induced by insulin in ATII cells. PI3K is a central signaling molecule in insulin action and the signaling transduction is mainly transmitted through its downstream target Akt [20]. Activation of Akt allows insulin- stimulated glucose uptake by inducing the translocation of type 4 glucose transporter [49]. These results confirmed that insulin-induced up-regulation of ENaC promoted AFC via activation of PI3K/Akt pathway, but this was contrasts with previous finding that Akt was not involved in the Na^+ ^transport by ENaC in distal renal tubule epithelial cells [50]. All three subunits(α, β, γ) of ENaC contain conserved PY motifs in the cytosolic COOH-terminal domain that interacted with WW domains 3 and 4 of Nedd4-2, which has been shown to negatively regulate ENaC expression in vitro and in vivo [29,51,52]. The binding of Nedd4-2 to these motifs results in internalization and degradation of ENaC due to ubiquitination [53-56]. The phosphorylation motif for Akt has been proved to be identified with a conserved PY motif, which provides a binding site for WW domains of Nedd4-2[57-59]. In this study, we found that insulin-induced decrease in the levels of Nedd4-2 in coimmunoprecipitated α-ENaC, β- ENaC and γ-ENaC were blocked with LY294002 and Akt inhibitor treatment respectively These findings indicated the effect of insulin on inhibition of Nedd4-2 binding to ENaC via PI3K/Akt pathway. Recent study of Fisher rat thyroid cell proved the regulation of α-, β- and γ-ENaC heterologously expressed via PI3K/Akt pathway by suppression of Nedd4-2[60]. In addtion, co-administration of Akt inhibitor and SGK1 inhibitor, reported to inhibit SGK1[61], significantly inhibited insulin-induced increase in the expressions of α-, β- and γ-ENaC, as well as increased the insulin-induced decrease in the expression of Nedd4-2 compared with administration of Akt inhibitor alone in ATII cells. These results supported the findings that regulation of ENaC by SGK1 via inhibition of Nedd4-2 previously reported [62,63]. Here, we focused on the role of insulin-induced Akt activation on ENaC.

In conclusion, the present data demonstrated that insulin alleviated pulmonary edema, enchaced AFC and attenuated lung injury in rats of LPS-induced ALI without affecting blood glucose levels. Activation of Akt, linking PI3K and insulin signaling pathway, is necessary and sufficient for increase in the expression of ENaC by inhibition of Nedd4-2.

## Abbreviations

Akt: protein kinase B; AFC: alveolar fluid clearance; ALI: acute lung injury; ARDS: acute respiratory distress syndrome; BALF: bronchoalveolar lavage fluid; ENaC: epithelial sodium channel; ELISA: enzyme-linked immunosorbent assay; IL-6: interleukin-6; LPS: lipopolysaccharide; MPO: myeloperoxidase; Nedd4-2: neuronal expressed developmentally downregulated 4-2; PI3K: phosphatidylinositol 3-kinase; SGK1: serum- and glucocorticoid-regulated protein kinase1; TNF-α: tumor necrosis factor-α

## Competing interests

The authors declare that they have no competing interests.

## Authors' contributions

WD and DXW participated in the conception and design of the study. WD and CYL performed the animal study, ELISA bronchoalveolar lavage fluid collection, lung histology, immunocytochemistry, RT-PCR and western blotting. WD, JT and WZ performed the cell culture RT-PCR and western blotting. WD analyzed the data and drafted the manuscript. DXW participated in the revision of the manuscript. All authors have read and approved the final manuscript.
